# Risk assessment of mixed and displacement ventilation (LAF) during orthopedic and trauma surgery on COVID-19 patients with increased release of infectious aerosols

**DOI:** 10.3205/dgkh000342

**Published:** 2020-05-14

**Authors:** Axel Kramer, Rüdiger Külpmann, Arnold Brunner, Michael Müller, Georgi Wassilew

**Affiliations:** 1 Institute of Hygiene and Environmental Medicine, University Medicine Greifswald, Germany; 2 Lucerne University of Applied Sciences and Arts, E & A, Horw, Switzerland; 3 Brunner Consulting, Pfaeffikon ZH, Switzerland; 4 Center for Musculoskeletal Surgery, Charité – University Medicine Berlin, Germany; 5 Clinic and Ambulance for Orthopedic and Orthopedic Surgery, University Medicine Greifswald, Germany

**Keywords:** Displacement ventilation, laminar air flow, mixed ventilation, no ventilation, negative pressure, SARS-CoV-2 spread

## Letter

### Potential transmission routes

The SARS-CoV-2 virus is primarily released from the respiratory tract as an aerosol, which can be increasingly released through intubation, bronchoscopy, or rhinoscopy, for example [1], [2]. Small particles can remain suspended in the air for several hours [2]. It is not known how long the corona virus persists in room air. On inanimate surfaces such as metal, glass, or plastic, human coronaviruses, e.g., Severe Acute Respiratory Syndrome (SARS) coronavirus, Middle East Respiratory Syndrome (MERS) coronavirus, or endemic human coronaviruses (HCoV) can persist for up to 9 days [3]. SARS-CoV-2 is also detectable in the blood, with a prevalence of 15% [4]. Thus, for everyone in the operating room (OR), various infection risks exist.

The patient’s head area can be covered during surgery, provided that the intervention does not target the head area. In contrast, during larger operations in orthopedics and trauma surgery, not only blood, bone, or tissue particles are distributed in the OR, but also blood dust created by a surgical hammer, for example, behave similarly to aerosols.

The role of vapors from electrocautery has not been established. Therefore, it should be avoided or an additional smoke extraction system should be used.

### Basic hygiene measures

Accordingly, all precautions must be taken to protect the personnel in the operating room, i.e., protective clothing, FFP3 mask, safety glasses, and if necessary foot protection or waterproof boots. After the finalization of the surgical operation, all surfaces in the OR must be disinfected with a virucidal disinfectant such as an oxygen releaser or hypochlorite. If a device for room disinfection with nebulizing hydrogen peroxide is available, it should either be used between operations (total processing time usually 65 minutes before re-entering in the OR). 

### Spatial separation

In general, a given OR should only be used for SARS-CoV-2 patients.

### Operation of ventilation systems

No evidence is available upon which to base a risk assessment of ventilation systems, but the following conclusions can be drawn from the results of the assessment of different room airflow patterns:

- ORs with laminar air flow (LAF) (in Germany room class Ia) have a significantly larger ventilation volume flow than operating theaters with mixed ventilation (in Germany room class Ib), which means that aerosol dilution in the operating room with LAF ventilation proceeds considerably faster. In operating rooms with LAF, additional protection for the surgical team and the patient is ensured by the directed instead of only mixed ventilation in the operating area. If the exhaust air from the OR flows directly to the outside via the exhaust-air device, the OR can be used without restrictions. The usual protective measures would be sufficient in the OR.
  However, if the neighboring rooms are ventilated with the “exhaust air” from the operating room using overflow technology, contamination of these rooms cannot be ruled out. Since this air is highly diluted and considering that in these operating theater concepts the supply corridor is excessively ventilated, we consider a general exclusion to be incommensurate. In such a case, we recommend an individual risk assessment.
- If the ventilation system can be switched to negative pressure, this ensures that no viruses from the operating room can infiltrate neighboring rooms by overflow. But opening doors immediately interrupts the negative pressure and allows air exchange with the surroundings. Therefore, the doors must be kept closed during the entire operation.
- Due to the mixed flow and the significantly lower ventilation volume with swirling of aerosols, operations in class Ib rooms pose a higher risk of contamination to the surgical team. It is questionable whether the FFP3 mask ensures a seal tight enough to exclude putting the team at risk. In this event, protection of the surgical team can be achieved by wearing overpressure body exhaust suits [5].
- ORs or intervention rooms of class II rooms with mechanical turbulent ventilation also have an air exchange rate and often are not equipped with sterile filters. Therefore, they are also not acceptable for surgery on COVID-19 patients.
- ORs without mechanical ventilation are also not acceptable, since released aerosols are not diluted and the highest aerosol concentration emerges through the opened doors at the end of the operation.

## Notes

### Competing interests

The authors declare that they have no competing interests.
